# Treatment of Involuntary Seminal Emissions by Compression

**Published:** 1846-05-01

**Authors:** 


*Treatment of Involuntary Seminal Emissions by Compression.*—Every practitioner has doubtless met with repeated instances of this affection, in which all efforts to relieve have proved fruitless, and the patient is left a prey to the hypochondriacal apprehensions which almost invariably attend it. The practice of Cauterization, introduced by Lallemand, appears to be successful in some cases; yet, to our knowledge, it is by no means an infallible remedy. We attribute its apparent efficacy, in a great measure, to revulsion, and the moral effect of the operation. There is much room for doubt that the Pathology of the affection consists chiefly in an irritable condition of the urethra. The truth is, it is a disorder involving different pathological, or physiological conditions. It may proceed from mere exuberance of the seminal secretion, and from excessive activity of the venereal appetite. These may induce it separately, or combined, without involving, strictly speaking, any pathological condition. On the other hand, it may proceed from a local irritability as a consequence of sexual excesses, or other abuses, a diseased state of the urethra, &c. With these views, it is not to be expected that any local remedies will prove effectual in all instances, and that in cases in which they are useful their operation is chiefly palliative, until other measures are successful, or the morbid habit is broken. We have been led to make these remarks from seeing in the New York Medical and Surgical Reporter a letter from Dr. Batchelder, of Utica, on the subject of compression on the perineum as a remedy for this complaint; in connection with which, the editor cites from Rankings' abstract on account of the same method practiced by M. Braschet of Paris. Dr. Batchelder states that he has been in the habit of using compression for more than twenty years. He regards it in the light of a palliative, keeping the complaint at bay until other means can be brought to bear against it. He does not describe his apparatus, but says that it closely resembles that of Braschet. The latter is described as follows: "The compressing bandage is formed of a leather belt, from behind which proceeds a thigh strap, at first single, and then bifurcated, to leave the genital organs free, and to be brought round and attached to the belt by two buckles with thongs. The thigh strap has a moveable pad in the middle, which is placed over the point to be compressed, and drawn as tightly as possible. This simple method differs essentially from the circular compression of the penis by rings, bands, on forceps—all of which are liable to serious accidents; the least of which is the repulsion of the semen towards the bladder, which occasions an illusory appearance of a cure, since, although the semen does not pass outward, it is not the less certainly evacuated; that is to say, expelled from its reservoirs."

This treatment is easily put into practice, and we imagine that some of our readers will be glad to make trial of it. It seems to us that the same apparatus would be applicable to emission of urine during sleep by children. If we mistake not, we have seen somewhere the same method advised for this troublesome difficulty.

